# Antigen-Specific Priming is Dispensable in Depletion of Apoptosis-Sensitive T Cells for GvHD Prophylaxis

**DOI:** 10.3389/fimmu.2014.00215

**Published:** 2014-05-19

**Authors:** Shai Yarkoni, Jerry Stein, Isaac Yaniv, Nadir Askenasy

**Affiliations:** ^1^Cellect Biomed, Kfar Saba, Israel; ^2^Bone Marrow Transplant Unit, Department of Pediatric Hematology-Oncology, Schneider Children’s Medical Center of Israel, Petah Tikva, Israel; ^3^Frankel Laboratory, Center for Stem Cell Research, Schneider Children’s Medical Center of Israel, Petah Tikva, Israel

**Keywords:** graft versus host disease, T cell depletion, apoptosis, Fas, TNF receptors, antigen-specific stimulation

## Abstract

Prophylactic approaches to graft versus host disease (GvHD) have employed both phenotypic reduction of T cells and selective elimination of host-primed donor T cells *in vitro* and *in vivo*. An additional approach to GvHD prophylaxis by functional depletion of apoptosis-sensitive donor T cells without host-specific sensitization *ex vivo* showed remarkable reduction in GHD incidence and severity. We address the role and significance of antigen-specific sensitization of donor T cells and discuss the mechanisms of functional T cell purging by apoptosis for GvHD prevention. Host-specific sensitization is dispensable because migration is antigen-independent and donor T cell sensitization is mediated by multiple and redundant mechanisms of presentation of major and minor histocompatibility complex and tissue antigens by donor and host antigen-presenting cells. Our data suggest that potential murine and human GvH effectors reside within subsets of preactivated T cells susceptible to negative regulation by apoptosis prior to encounter of and sensitization to specific antigens.

## Introduction

Donor T cells play a dual role in transplantation of hematopoietic stem and progenitor cells (HSPC). On the one hand, they mediate potentially lethal graft versus host disease (GvHD) (1), therefore the most effective approach to GvHD prophylaxis is transplantation of large number of purified hematopoietic progenitors that overcome antigenic barriers (2, 3). On the other hand, T cells support engraftment, improve resistance to infections, and contribute to graft versus tumor (GvT) reactivity (4–6). The significance of donor T cells in facilitation of engraftment is emphasized by superior outcome of T cell-replete grafts as compared to purified progenitors, and the inverse relationship between the number of grafted donor T cells and transplant-related mortality (7, 8). Initial studies of selective phenotypic depletion of T cell subsets have shown limited efficacy in GvHD prevention, emphasizing the capacity and participation of multiple immune-reactive species (9). Doses of 10^4^–10^5^ CD3^+^ T cells/kg along significant number of hematopoietic progenitors (~10^7^ CD34^+^/kg) are considered as threshold conditions that support engraftment at reduced risk of high grade GvHD in matched and mismatched unrelated transplants (10). However, one of the difficulties of fractional phenotypic depletion is the poor correlation between the number of donor T cells and severity of the GvH reaction due to indiscriminate selection of mediators of inflammation (11), as exemplified by vigorous GvHD elicited by antigen-inexperienced T cells in umbilical cord blood (UCB) (12).

## *Ex vivo* Depletion of Host-Primed Donor T Cells

**Figure 1:**
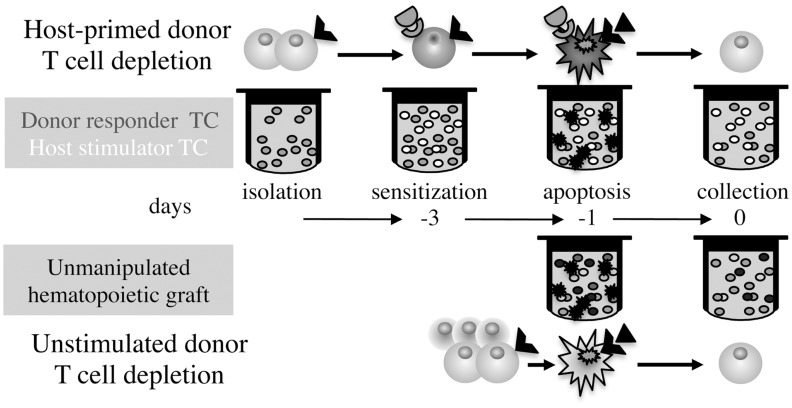
**Differential time axis and procedures for GvHD prophylaxis**. *Ex vivo* simulation of GvHD by exposure of isolated donor T cells to irradiated host stimulators followed by depletion of the sensitized T cells, as compared to elimination of apoptosis-sensitive donor T cells in whole grafts without antigen-specific stimulation.

An alternative effective approach to GvHD prophylaxis is *ex vivo* stimulation of alloreactivity by exposure of donor T cells to host antigens and depletion of the reactive responders, a conceptual frame that awards dual selectivity: responsiveness to host antigens of a fraction of donor clones and selective depletion restricted to activated T cells (Figure 1). Characteristics of T cell activation targeted for selective depletion include fast-cycling (13), sensitivity to fludarabine (14) metabolic mitochondrial activity (15), and photoactivation of synthetic psoralen (16). Superior outcome attained by depletion of the α chain CD25 IL-2 receptor (IL-2R) in conjunction with CD69 (17) and CD71 (transferrin receptor) (18) emphasizes phenotypic variability of activated T cells where neither one can be considered as universal marker of activation. IL-2R is an attractive target of activation because internalization of the receptor/ligand complex introduces toxic moieties, such as IL-2R monoclonal antibodies conjugated to ricin and diphtheria toxins (19, 20), and IL-2 fusion proteins encoding apoptotic moieties such as caspase-3 (21). A fundamental characteristic of immune cell activation is upregulation of TNF family receptors rendering them susceptible to negative regulation by activation-induced cell death (AICD), where Fas cross-linking by membrane-bound Fas-ligand (FasL) is the common executioner of apoptosis (22). *Ex vivo* depletion of host-sensitized donor T cells with agonistic Fas antibodies (23), cross-linking by soluble FasL oligomers (24), and expression of the ligand in dendritic cells (DC) (25) in murine models and human mobilized peripheral blood (MPB) cells (26) has reduced GvHD severity.

All procedures of fractional depletion of host-primed donor T cells have documented significant advantages of add back of insensitive T cells: support engraftment, sustain reactivity against tumors (24), and infections in the early post-transplant period (26), due to persistence of effector/memory cells that are relatively insensitive to AICD (22). However, the main drawback of this technique is the relatively slow sensitization process that requires mixed lymphocyte cultures of ~3 days, imposing laborious isolation of T cells and cryopreservation of progenitors. Because transduction of apoptotic signals is very effective, this approach to GvHD prophylaxis has been improved through increased proficiency of stimulation using non-selective T cell stimulation with CD3 antibodies (23), and DC to amplify antigen presentation (25) and boost T cell proliferation (13). Although GvH simulation by donor T cell sensitization to the host is intuitive, it has been long recognized that cytotoxic T cell assays *in vitro* correlate poorly with GvH reactivity against minor antigens *in vivo* (27), possibly because gradual transition to apoptosis-insensitive effector/memory phenotypes in culture may cause persistent recollection of alloresponses in residual T cells. Early post-transplant administration of cytotoxic agents such as cyclophosphamide may be more effective in concomitant suppression of reciprocal sensitization of donor GvH effectors and host versus graft (HvG) rejection (28).

## *Ex vivo* T Cell Depletion Without Host-Specific Sensitization

The GvH reaction is effectively prevented, on the one hand, by non-selective depletion of donor T cells using phenotypic markers (9), and on the other hand, by selective depletion of host-primed donor T cells (13–21, 23–26). We reasoned that elimination of apoptosis-sensitive donor T cells without host-specific priming may be effective in GvHD prevention. Exposure of murine splenocytes and bone marrow cells (BMC) to FasL reduced significantly the clinical and histological GvHD indices and improved survival following cytokine storm induced by lipopolysaccharide (LPS) in haploidentical transplant models (29). Residual donor T cells retained the major *in vivo* activities that commend their inclusion in the graft: sustained reactivity against solid tumors and haematological malignancies, and support of progenitor engraftment when co-administered with the graft and as delayed donor lymphocyte infusion. Reduced GvHD severity was validated in xenochimeric mice grafted with human MPB exposed to FasL and TNFα *ex vivo* for short periods of time, showing apoptotic death of T and B lymphocytes and myeloid cells, decreased propensity of activation markers in viable T cells, and sustained reactivity against tumors (30).

The short incubation period in this procedure (hours) over depletion of host-primed T cells (days), and obviation of T cell isolation and cryopreservation of progenitors, associated with loss of significant fractions in the freezing/thawing process, are of prime significance (Figure 1). Murine and human hematopoietic progenitors share innate resistance to apoptotic signaling triggered by the TNF family receptors *in vitro* and *in vivo*, which transduce trophic signals and improve the efficiency of engraftment and shorten the tempo of reconstitution (31–35). In fact, trophic signals are transduced by the same receptors that mediate AICD in human immune cells, including Fas, TNF-R1, and TRAIL-R1, thus both GvH prevention and progenitor stimulation are simultaneously attained by pretransplant exposure to the cognate ligands.

Most approaches based on different techniques of selective elimination of activated T cells following simulation of host-specific priming *ex vivo*, as well as phenotypic depletion of T cell subsets have shown beneficial effects on GvHD. Similar efficacy of antigen-dependent and independent fractional deletion of apoptosis-sensitive T cells imposes two related questions: what is the significance of host antigen-specific sensitization of donor T cells for GvHD prophylaxis and how does elimination of unstimulated T cells ameliorate this reaction. The following discussion attempts to deduce some of the characteristics of GvHD effectors.

## Quantitative Aspects of Apoptotic T Cell Depletion

Application of an apoptotic challenge to cultures of splenocytes from naïve mice housed in a barrier facility results in depletion of ~50% T cells and commensurate elimination of CD4^+^CD25^+^FoxP3^+^ naturally occurring regulatory T cells (36). Fractional apoptosis is markedly lower (40%) in mixed cultures as compared to isolated T cell preparations (70%) (37), due to cytokine deprivation (IL-2) and modulation of T cell viability by T cell receptor (TCR)-associated CD3 signaling and CD28 co-stimulation (37). Consequently, the number of residual T cells following an apoptotic challenge are significantly higher in hematopoietic grafts than isolated T cell suspensions, yet similar protective effects were obtained by purging of host-primed and antigen-inexperienced T cells (29). Significant decline (~2.5-fold) in GvHD severity was attained by the apoptotic challenge in murine haploidentical transplants, corresponding to 2 × 10^8^ viable T cells/kg (29). Likewise, GvHD was reduced in xenogeneic transplants of MPB cells at doses of 1.5 × 10^8^ viable T cells/kg, which represent almost 3-log higher numbers than the recommended doses of unmanipulated donor T cells in mismatched transplants (4–6, 10). Even megadoses of 10^7^ progenitors/kg (2) can be safely administered as T cell-replete grafts following exposure to the apoptotic challenge, provided that the progenitor:T cell ratio is below 1:20. Evidently, the duration of graft preparation is determined by the differential sensitivities of T cells from various origins: UCB-derived T cells are relatively resistant to 48 h of exposure to death ligands, whereas 40–50% of T cells in BM and MPB are depleted within 18–32 and 4–8 h, respectively (30, 32, 34, 38). The duration of these cultures is shorter than the critical period of 48 h associated with significant decline in efficiency of engraftment (39, 40).

## Qualitative Aspects of T Cell Depletion

GvHD prophylaxis could not be attributed to selective depletion of particular T cell subsets in our studies, which may essentially represent the most significant advantage. At the first level, exposure of murine splenocytes and BMC to apoptotic signals without host-specific priming results in balanced reduction in CD4^+^ and CD8^+^ T cells, each one having the capacity to mediate experimental and clinical GvHD (41–43). At the second level, functional depletion by apoptosis affects all immune cells within the graft including professional antigen-presenting cells (APC) and disrupts the activation cascades at multiple levels (22). For example, B lymphocytes and myeloid cells endowed with antigen-presenting capacity are generally more sensitive to apoptosis than unstimulated T cells in hematopoietic grafts derived from UCB, BM, and MPB (30, 32–35, 38). At the third level, the apoptotic challenge particularly but incompletely removes T cells expressing activation markers such as CD25 and CD69 (29, 30), which impact experimental (17, 18) and clinical GvHD (44). An interesting observation was modulation of the immune responses of recipients of mismatched T cells preexposed to an apoptotic challenge, which displayed intact responses to alloantigens *in vitro* and similar rates of immune reconstitution as recipients of unmanipulated T cells (29). Survival of 70% recipients of apoptosis-treated T cells following LPS was associated with reduced proliferative responses of host splenocytes as compared to medium-incubated controls that universally succumbed to lethal GvHD. Since this phenomenon was observed in a non-engrafting GvHD model of adoptive T cell transfer following sublethal irradiation, donor lymphocytes evidently reduced the responsiveness of host splenocytes to cytokine-mediated mitogenic stimulation.

## Host-Specific Priming is Dispensable in GvHD Prophylaxis

The pathophysiology of GvHD involves multiple pathways of T cell migration to tissues and lymph nodes, sensitization by professional and non-professional APC against physiological host alloantigens and tissue epitopes exposed by conditioning-mediated tissue injury, and amplification of cytotoxic activity by cytokines. CD8^+^ T cells have been initially considered as the culprit mediators of GvHD, but functional variability may be caused by the differential requirements for direct engagement of tissue antigens by CD8^+^ but not CD4^+^ T cells (45). We will briefly consider several features of the GvH reaction, without addressing migration because T cells exposed to an apoptotic challenge were shown to navigate effectively to target tissues and regional lymph nodes (29).

From the immunological point of view, dual sensitization within the tissue and regional lymph nodes ensures efficient sensitization with redundant and synergistic consequences (46–48). Allogeneic transplants are characterized by mixed chimerism of professional APC, though grafted T cells are primed by APC of donor (49) and host origin (50), with decisive inductive activity of tissue-resident APC (46, 51). Although major histocompatibility complex (MHC) disparity is generally associated with vigorous GvH reactions (52), alloresponses are restricted to a limited number of donor CD4^+^ and CD8^+^ T cell clones with selective and compatible TCR rearrangement (52–54), and cytotoxic cells frequently target minor histocompatibility antigenic repertoires (miHA) (55–57). Effector/memory T cells are less effective mediators of acute GvHD as compared to naïve T cells (58, 59), however, their continued presence promotes persistent acute and chronic GvH reactivity (46, 60). The apparent sequence of events implies that donor T cells migrate in an antigen-independent manner to tissues and lymphoid organs and can be primed at both sites by diverse subsets of APC. Potent and redundant pathways of antigen recognition include systemic indirect and direct presentation of MHC and miHA by donor and host APC, respectively, and *in situ* instruction by resident APC in the target tissues. Clinical presentation of GvHD in tissues most sensitive to injury by preparative conditioning, bone marrow, intestine, and skin suggests that damage of these proliferative target tissues plays a role in the process of acute sensitization of GvHD effectors. Currently there is no positive characterization of a particular T cell subset, TCR configuration, and mechanism of cytokine exacerbation that accounts solely for induction and propagation of the GvHD reaction. In addition to the multiple redundant mechanisms of activation of diverse subsets of GvHD effectors, initiation of inflammation and execution of injury to the target tissue are inflicted by numerous cytotoxic pathways.

Several scenarios have been proposed to account for variations in all these parameters using different experimental models that frequently use transgenes lacking particular molecules, which are difficult to interpret and underestimate the involvement of compensatory mechanisms. For example, host APC activate donor CD8^+^ T cells by direct miHA presentation in the context of class I MHC (50), and donor APC process host miHA of non-hematopoietic tissues as foreign antigens and present to CD4^+^ T cells in the context of class II MHC (55). Another possibility is sequential direct and indirect antigen presentation by host and donor APC, respectively, suggesting that donor APC amplify GvH reactions initiated by host APC (61). An additional scenario suggests direct miHA targeting by cytotoxic T cells, with MHC disparity determining the intensity of the inflammatory reaction (62).

The elaborate mechanisms of sensitization of multiple T cells subsets explain the capacity of multiple deletional approaches to restrain GvH reactivity, including highly selective depletion of host-primed T cells and also antigen non-specific lymphoreduction. For example, the risk of GvHD is reduced by fractional depletion of naïve human CD45RA^+^ lymphocytes with persistent responsiveness to infectious agents (63), and by non-specific immunomodulation of the donors with complete and incomplete Freund adjuvant and Toll-like receptor activation with CpG motifs without impairing GvT reactivity (64). Donor T cells may generally be less reactive to alloantigens under these conditions, but it is also possible that activated T cells underwent excessive deletion by apoptosis after transplantation into partially immunosuppressed recipients. Evidence of this mechanism evolves from reduced GvHD severity following pretransplant antigen non-specific stimulation of donor T cells with agonistic anti-CD3 antibodies (65), a counter-intuitive approach because T cell sensitization with CD3/CD28 generally enhances both GvH and GvT reactions (66). The apparent mechanism is effective purging of hyperactivated donor T cells susceptible to AICD in radiation chimeras, though it is yet undetermined whether deletion occurred in the recipient prior to or following specific sensitization to host antigens. Importantly, these deletional approaches to GvHD prophylaxis neither impaired facilitation of progenitor engraftment nor GvT reactivity, which are often dissociated in the transplant setting (4–6).

## Who are the Candidate GvHD Effectors?

GvH is an acute physiological immune reaction against foreign antigens mediated by mature donor T cells that mirrors HvG rejection and elicits complex cascades of activation involving multiple redundant mechanisms of antigen presentation and cytokine circuits. Delayed clinical appearance of GvHD by a period of several weeks follows progressive tissue damage inflicted by inflammation and is frequently associated with infection, which may trigger and intensify GvH and reciprocally, GvH-mediated injury perpetuates infection by disruption of the mucosal barriers. Effector/memory (46, 58–60), naïve (63), stimulated (66) CD4^+^ and CD8^+^ T cell subsets (41–43, 45) that display high metabolic activity (15), fast proliferation (13, 14), activation markers (17–21), and sensitivity to apoptosis (23–26, 29, 30) can elicit GvH reactions (9) following recognition of major and minor histocompatibility and tissue antigens (47, 48, 56) introduced by donor and host cells with antigen-presenting capacity (46, 49–51). Therefore, early onset of the GvH reaction and redundant activity of multiple cell types is consistent with reduced inflammation by preemptive depletion of apoptosis-sensitive T cells, one of the unequivocal signs of activation. Although apoptosis-resistant donor T cells display intact responses to allosensitization and stimulation *in vitro* (36), they yield quite restrained responses in the context of GvH reactivity *in vivo* (29, 30). Concomitant depletion of all lineages of apoptosis-sensitive immune cells from the graft also reduces the capacity of antigen presentation and elaboration of inflammatory cytokines (22). The mechanistic insight evolving from amelioration of GvHD by depletion of apoptosis-sensitive T cells without antigen specificity is a significant involvement of proactive immune cells susceptible to negative regulation in this immune reaction.

It will be imperative to monitor the phenotypes of depleted and residual T cells and the responses to host alloantigens in the clinical setting under various conditioning protocols and in association with prevalent infections in the complex clinical setting. It will be interesting to determine whether fractional depletion of unstimulated donor immune cells further protects from GvHD by polarizing the sensitivity to apoptosis using IL-2 to preserve regulatory T cells (21, 36, 37). In view of the potential of UCB-derived T cells to elicit potent GvH reactions (12) and insensitivity of these naïve cells to apoptosis under unstimulated conditions (38), AICD may be achieved on a shorter time scale using various antigen-non-specific stimuli (67–69). It remains to be determined whether the apoptosis-mediated approach to GvHD restrains chronic disease, which is less characterized and largely unresponsive to immunosuppressive therapy.

## Concluding Remarks

Although the approach of donor T cell sensitization against the host to simulate GvH *ex vivo* is intuitive, it has been long recognized that cytotoxic T cell assays *in vitro* cannot predict and do not correlate with GvH reactivity against host antigens *in vivo* (32). The poor correlation between number of T cells and GvHD intensity (11) shifts the attention to the quality of T cells included in donor inoculum: host-selective priming is dispensable suggesting that GvHD effectors reside within activated subsets of donor T cells.

## Conflict of Interest Statement

Shai Yarkoni serves as CEO of Cellect Biomed and has equity in this company. The other co-authors report no conflicts of interest.
